# Effect of Probiotics on the Symptomatology of Autism Spectrum Disorder and/or Attention Deficit/Hyperactivity Disorder in Children and Adolescents: Pilot Study

**DOI:** 10.1007/s10802-024-01278-7

**Published:** 2025-01-11

**Authors:** Meritxell Rojo-Marticella, Victoria Arija, Josefa Canals-Sans

**Affiliations:** 1 Nutrition and Mental Health Research Group (NUTRISAM), Universitat Rovira I Virgili (URV), Carretera de Valls, S/N, 43007 Tarragona, Spain; 2 Department of Psychology, Research Center for Behavioral Assessment (CRAMC), Universitat Rovira I Virgili (URV), Tarragona, Spain; 3 Pere Virgili Institute for Health Research (IISPV), Universitat Rovira I Virgili (URV), Reus, Spain

**Keywords:** Probiotics, Autism spectrum disorder, Attention deficit/hyperactive disorder, Children, Adolescents, Randomized controlled trials

## Abstract

The aim of this study is to investigate the impact of using probiotics with strains related to dopamine and gamma-aminobutyric acid production on clinical features of autism spectrum disorder (ASD) and/or attention deficit/hyperactivity disorder (ADHD). This randomized, controlled trial involved 38 children with ADHD and 42 children with ASD, aged 5–16 years, who received probiotics (*Lactiplantibacillus plantarum* and *Levilactobacillus brevis* 109/cfu/daily) or placebo for 12 weeks. Parent-reported symptoms were assessed using Conners' 3rd-Ed and the Social Responsiveness Scale Test, 2nd-Ed (SRS-2), and children completed the Conners Continuous Performance Test, 3rd-Ed (CPT 3) or Conners Kiddie CPT, 2nd-Ed (K-CPT 2). Executive functions, quality of life and sleep patterns were also parent-assessed. Intention-to-treat analyses, controlling for sociodemographic and nutritional covariates, revealed no significant inter-group differences in parent-reported or neuropsychological data after the probiotic intervention. However, age-stratified analyses showed improved hyperactivity-impulsivity symptoms in younger children with ASD (Cohen’s d = 1.245) and ADHD (Cohen’s d = 0.692). Intra-group analyses supported these findings in the aforementioned age and intervention group for both diagnoses. An improvement in impulsivity for children with ASD was also observed in the intra-group analysis of the CPT commissions scores (probiotic: p = 0.001, Cohen’s d = -1.216; placebo: p = 0.013, Cohen’s d = -0.721). A better comfort score (quality of life) was shown in children with ASD (probiotic: p = 0.010, Cohen’s d = 0.722; placebo: p = 0.099, Cohen’s d = 0.456). The probiotics used, may improve hyperactivity-impulsivity in children with ASD or/and ADHD and quality of life in children with ASD. Further research is warranted to explore probiotics as an adjunctive therapeutic intervention for NDs.

Trial registration: clinicaltrials.gov Identifier: NCT05167110.

**Supplementary Information:**

The online version contains supplementary material available at 10.1007/s10802-024-01278-7.

## Introduction

Neurodevelopmental disorders (NDs) are described by the World Health Organization (WHO) as behavioral and cognitive disorders that emerge during the developmental period and persist throughout life, leading to significant difficulties in specific intellectual, motor, neuropsychological, communicative, or social functions (World Health Organization, 2019). Among the more prevalent NDs are autism spectrum disorder (ASD) (Zeidan et al., 2022) and attention deficit/hyperactivity disorder (ADHD) (Sayal et al., 2018). In the Spanish school population, the estimated overall prevalence of ASD is 1.53% (Morales-Hidalgo et al., 2021), while ADHD has a prevalence of 5.4% (Canals-Sans et al., 2021).

ASD is characterized by social interaction and communication difficulties, as well as repetitive and restrictive patterns (American Psychiatric Association, 2013). ADHD, on the other hand, is primarily characterized by symptoms of inattention, hyperactivity, and impulsivity, which can manifest in three different presentations: inattentive, hyperactive/impulsive, or combined (American Psychiatric Association, 2013). Both ASD and ADHD often involve deficits in executive functioning, which is crucial for cognitive, behavioral, and emotional control processes in children (Kofler et al., 2019; Otterman et al., 2019).

The etiology of ASD and ADHD is multifactorial, involving environmental, biological, and genetic risk factors. There is a high comorbidity between ASD and ADHD at the clinical level (Dellapiazza et al., 2021; Rau et al., 2020), and evidence suggests shared neural network patterns (Harikumar et al., 2021) and genetic overlap between the two disorders (Mariggiò et al., 2021). Specific genetic polymorphisms have been identified as potential contributors to this genetic overlap, affecting neurodevelopment, neurotransmission, and synaptic connectivity processes. Variations in genes associated with dopamine and serotonin have been implicated in both ASD and ADHD, as well as alterations in genes involved in the inhibitory neurotransmitter-gamma-aminobutyric acid (GABA) pathways, reducing its levels and providing worse inhibitory control, which are commonly observed in both disorders. (Bollmann et al., 2015; Mishra et al., 2022; Port et al., 2017; Purkayastha et al., 2015; Puts et al., 2020).

### Neurodevelopmental Disorders and Gut-Brain Axis

Ongoing research is investigating new etiopathogenic factors for ASD and ADHD, with a particular focus on the relationship between the nervous system and the gastrointestinal tract. This connection is mediated by the gut-brain axis, which facilitates bidirectional communication between the central nervous system (CNS), enteric nervous system, and gut microbiota (Cerdó et al., 2017). Recently, there has been growing interest in exploring the potential role of gut microbiota and its metabolites as contributing factors in various human conditions, including NDs (Barrio et al., 2022). Certain bacterial strains of species of *Bifidobacterium* and *Lactobacillus*, such as *L. brevis*, *B. dentium*, *L. plantarum* and *L. paracasei*, can participate in the synthesis and control of the release of neurotransmitters like acetylcholine, dopamine, serotonin and GABA and its precursors in certain gut cells, like enterochromaffin cells (Barrett et al., 2012; Cheng et al., 2019; Dash et al., 2022; Hamamah et al., 2022).

Emerging evidence from several studies suggests that individuals with ASD and/or ADHD sometimes exhibit gut microbiota dysbiosis, which is an alteration either of its composition and/or abundance of some microorganisms that compose it (Aarts et al., 2017; Boonchooduang et al., 2020; Checa-Ros et al., 2021; Gkougka et al., 2022; Iglesias–-Vázquez et al., 2020; Shirvani-Rad et al., 2022; Sukmajaya et al., 2021). This dysbiosis may influence the production of neurotransmitters, potentially affecting symptom severity and overall well-being. *Bifidobacterium* has been found to be elevated in adults and children with ADHD, which may lead to impaired dopamine-related functions in the CNS (Shirvani-Rad et al., 2022). In contrast, lower percentage of *Bifidobacterium* in the composition of gut microbiota was found in children with ASD than in controls (Iglesias–Vázquez et al., 2020). In turn, another metagenomic study has shown that children and adolescents with ASD and ADHD share gut microbiota profiles (Bundgaard-Nielsen et al., 2023). While the study of this dysbiosis has so far been limited to bacterial microbiota, a recent study has extended metagenomic sequencing to new kingdoms in children with ASD, which has enabled the identification of very accurate markers in diagnosing autism condition (Su et al., 2024).

### Previous Probiotic Interventions

In relation to this previous literature, researchers have explored the potential of modulating gut microbiota through nutritional interventions, specifically probiotic supplementation, to restore balance and improve symptoms in individuals with ASD and ADHD. Probiotics can serve as an adjunctive treatment for NDs because they often target the same neurological pathways as conventional pharmacotherapy used for ADHD. However, probiotics are generally more user-friendly and have fewer side effect (Van Vyve et al., 2024). Systematic reviews reveal various study designs, including open-label controlled trials, cross-sectional studies with washout periods, and randomized controlled trials. Intervention periods range from three weeks to several months and involve different probiotic strains and measurement criteria (Amadi et al., 2022; Kang et al., 2018; Khanna et al., 2022; Ng et al., 2019; Rianda et al., 2019; Sivamaruthi et al., 2020; Vasiliu, 2023; L. J. Wang et al., 2022). The methodological differences between the studies and the variability in the composition of the gut microbiota itself could have conditioned the results of the interventions.

The literature on probiotic interventions in ASD is more extensive than that for ADHD. Some studies report positive effects on clinical symptomatology and behavior in ASD, noting improvements in autism severity, attention capacity, social affect, and core symptoms in children (Adams et al., 2011; Grossi et al., 2016; He et al., 2023; Liu et al., 2019; Parracho et al., 2010; Santocchi et al., 2016; Schmitt et al., 2023; Shaaban et al., 2017) and in adolescents as well (Mensi et al., 2021). Additionally, several authors have shown improvements on physiological characteristics common in children with ASD, such as GI symptoms and stool consistency (Grossi et al., 2016; Parracho et al., 2010; Santocchi et al., 2016; Shaaban et al., 2017). Specifically, after probiotic supplementation with *Lactobacillus acidophilus, Lactobacillus rhamnosus* and *Bifidobacteria longum,* Shaaban et al., (2017) found significant improvements in the severity of autism compared to the baseline evaluated at the start of the study. Nevertheless, Liu et al (2019) showed that an intervention using *Lactobacillus plantarum* in boys with ASD in comparison with placebo group, ameliorated secondary outcomes (inattention, hyperactivity-impulsivity and opposition-defiance behaviors) but not core symptoms of ASD. A recent systematic review and meta-analysis concluded that probiotics improve overall ASD symptomatology when combinations of strains are used, although this finding does not extend to core ASD symptoms such as restricted repetitive behaviors and social or communication issues in children (Lee et al., 2024). However, not all studies report positive results in improving psychological symptoms (Barba-Vila et al., 2024). In contrast, probiotic interventions in children with ADHD have been less extensively investigated. Results vary depending on study characteristics, such as sample age, primary objectives, and outcome measures. Some narrative reviews, report reductions in inattention and hyperactive/impulsive symptoms while others report improvements in physical, emotional and social characteristics, as well as better functioning at school, after interventions with probiotics with strains of *Lactobacillus,* mostly *rhamnosus* in its formulations (Checa-Ros et al., 2021; Pinto et al., 2022). However, the review of Kalenik et al., (2021) argue that there is still too little evidence to recommend the use of probiotics in children with ADHD. Most recently, Duarte Luiz et al., (2023) indicated that randomized controlled trials in both animal models and humans with *Lactiplantibacillus plantarum* showed improvements in ADHD symptoms and other neurological disorders. A recent systematic review concludes that probiotics can be a beneficial adjunctive treatment for ADHD in children, although it should be noted that many studies included methylphenidate alongside probiotic supplementation (Nahidi et al., 2024).

### Purpose of Current Study

Given the heterogeneous results from previous studies on children with ASD and/or ADHD, which suggest that certain strains of *Lactobacillus plantarum* may improve symptoms of both disorders, this study aims to investigate the impact of a nutritional intervention using the probiotic strains *Lactiplantibacillus plantarum* and *Levilactobacillus brevis* on the clinical characteristics of children with ASD and/or ADHD, as assessed by behavioral and neuropsychological tests. These strains are known for their role in synthesizing neurotransmitters such as GABA and dopamine. We hypothesize that supplementation with both probiotic strains may lead to an improvement in ASD and/or ADHD symptom severity and health-related quality of life.

## Methods

### Study Design and Participants

**Fig. 1:**
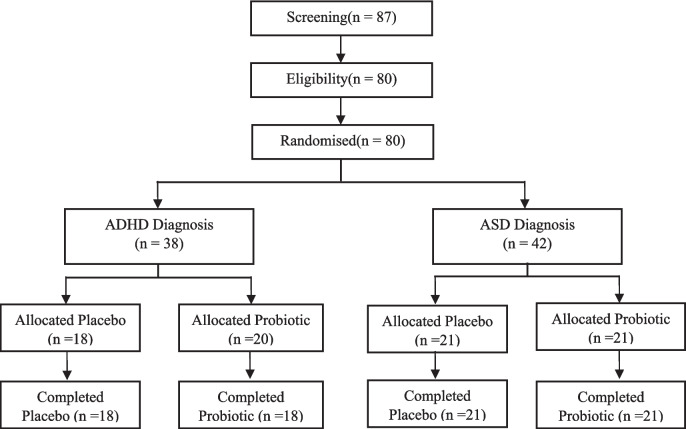
Flowchart of the participants in the study

The present study employed a randomized, double-blind, placebo-controlled trial design with two parallel arms using dietary supplements (Registry: ClinicalTrials.gov Identifier: NCT05167110). The study protocol was approved by the Institut d'Investigació Sanitària Pere Virgili (IISPV) drug research ethics committee (Ref.CEIM:030/2017). Figure 1 illustrates the flowchart of the participants in the study.

The sample consisted of 38 children diagnosed with ADHD and 42 children diagnosed with ASD, all meeting the Diagnostic and Statistical Manual of Mental Disorders, Fifth Edition (DSM-5) criteria. Participants with both diagnoses, ASD and ADHD (n = 11), were placed in the ASD group, considering this as the main diagnosis. Mental health professionals administrated the Autism Diagnostic Observation Schedule, Second Version (ADOS-2) (Lord et al., 2012) to the children and the Autism Diagnostic Interview-Revised (ADI-R) (Rutter et al., 2003) to the parents to collect information for ASD. Participants were recruited from specialized clinical centers for NDs in the province of Tarragona, Spain, as well as schoolchildren diagnosed through the EPINED project at Universitat Rovira i Virgili (Ref. PSI2015-64837-P and RTI2018-097124-B-I00) (Canals Sans et al., 2021; Morales-Hidalgo et al., 2021).

Children between 5 and 16 years old diagnosed with ASD and ADHD meeting DSM-5 diagnostic criteria were invited to participate in the study if their parents were willing to grant informed consent. Children were ineligible if they had had previous experience of adverse effects related to probiotic administration; had used probiotics within three months prior to the start of the intervention; had any medical conditions contraindicated for probiotic administration such as immunodeficiency, inflammatory bowel disease, chronic diarrhea or short bowel syndrome; had any intolerances or allergies to the treatment excipient (probiotic or placebo); or were undergoing ongoing antibiotic use at the start of the intervention.

### Procedures

Parents or guardians of children diagnosed with ASD and ADHD were contacted through the aforementioned clinical centers and provided with an informational leaflet about the study. Those who expressed an interest in participating were invited for an initial visit prior to the intervention, during which the project was further explained, and the parents/guardians signed the informed consent form.

During the first visit in the intervention phase, parents completed validated questionnaires regarding the clinical and health characteristics of their children. Simultaneously, the children underwent either the computerized Conners Continuous Performance Test 3rd Edition (CPT 3) or the Conners Kiddie Continuous Performance 2nd Edition (K-CPT 2), depending on their age.

After randomization, the research team provided the study product and detailed instructions for its administration to the participating parents or guardians. In the sixth week, a telephone follow-up was conducted to ensure adherence to the intervention and to address any potential concerns or issues. Families were also encouraged to reach out to the research team via email or telephone if they had any questions or needed support at any time throughout the intervention period.

At the end of the intervention (during the 12th-13th week), the parents completed the clinical questionnaires, and the children underwent the CPT 3 or K-CPT 2 once again.

### Nutritional Intervention

The active product used in this trial consisted of bacterial species that have been granted Qualified Presumption of Safety (QPS) status by the European Food Safety Authority (EFSA), indicating their safety for human consumption.

Participants were randomly assigned to either receive the *Lactiplantibacillus* mixture or placebo sachets for a period of 12 weeks. The *Lactiplantibacillus* sachets contained a bacterial mixture weighing 50 mg, which included 1 × 10^9^ CFU (colony-forming units) of *Lactiplantibacillus plantarum* CECT7485 (KABP023) and *Levilactobacillus brevis* CECT7480 (KABP052) in a 1:1 ratio. Each sachet also contained 0.01 mg of vitamin D, 266.49 mg of maltodextrin, 1623 mg of anhydrous dextrose, 50 mg of raspberry flavor, 10 mg of Silicon Dioxide E-551, and 0.5 mg of fructose. The placebo sachets contained 316.5 mg of maltodextrin, 1623 mg of anhydrous dextrose, 50 mg of raspberry flavor, 10 mg of Silicon Dioxide E-551, and 0.5 mg of fructose. Probiotic and placebo sachets had the same appearance, and were provided in anonymous, numbered boxes.

Both the researchers and the participants were unaware as to the allocation of the probiotic or the placebo intervention. The group assignment of each participant was revealed upon conclusion of the trial.

### Primary Endpoints: Psychological Assessment

The parents or guardians of the participating children with ASD and/or ADHD completed the following psychological tests both before and after the intervention:

- The Spanish version of the Conners 3rd Edition–Parent Short Form (K. C. Conners, 2008): This test assesses various domains including inattention, hyperactivity-impulsivity, learning problems, executive functioning, aggression/defiance, and peer relations through 45 items. T-scores were used for each scale, with scores ≥ 70 indicating very elevated levels, scores between 65 and 69 denoting elevated levels, scores between 60 and 64 representing high average levels, and scores ≤ 60 indicating average levels. Inattention and hyperactivity-impulsivity subscales have been considered as primary endpoints.
- Parents of children with ASD answered the Spanish version of the Social Responsive Scale-second edition (SRS-2) (Constantino & Gruber, 2012): This test evaluates the severity of ASD symptoms in natural settings using 65 items. It provides a global score as well as scores for scales assessing social awareness, social cognition, social communication, social motivation, and restrictive interests and repetitive behaviors. Severity level T-scores were used, with scores ≥ 76 indicating severe symptoms, scores between 66 and 74 indicating moderate symptoms, scores between 60 and 65 representing mild symptoms, and scores between 45 and 59 indicating normal functioning. Restrictive interests and repetitive behaviors, social communication and interaction and total score subscales have been considered as primary endpoints.

- Children over 8 years old were administered the Conners Continuous Performance Test 3rd Edition (CPT 3) (C. K. Conners, 1994a), and children between 4 and 7 years old were administered the Conners Kiddie Continuous Performance Test 2nd Edition (K-CPT 2) (C. K. Conners, 1994b). This test assesses problems related to inattentiveness, impulsivity, sustained attention, and vigilance. T-scores were used for each variable and measure, including detectability, error type variables (omissions, commissions, perseverations), and reaction time (RT) (Hit RT, HRT SD, variability, HRT block change, HRT ISI change). For HRT-related variables, scores ≥ 76 indicated atypically slow performance, scores between 60 and 69 indicated slow performance, scores between 55 and 59 indicated slightly slow performance, scores between 45 and 54 indicated average performance, scores between 40 and 44 indicated slightly fast performance, and scores below 40 indicated atypically fast performance. For all other variables, scores above 59 suggested elevated levels indicative of problems, with scores ≥ 70 indicating very elevated levels, scores between 60 and 69 indicating elevated levels, scores between 55 and 59 indicating high average levels, scores between 45 and 54 indicating average levels, and scores below 45 indicating low levels. Additionally, the software provided atypical T-score patterns indicating problems with inattentiveness, impulsivity, sustained attention, and vigilance, categorized into three severity ranges: strong indication, some indication, or no indication.

### Secondary Endpoints: Psychological Characteristics, Health-Related Quality of Life, Sleep Habits

The following secondary endpoints were answered by the parents and administered before and after the intervention:

- Learning problems, executive functioning and aggression/defiance of the Conners’ test.
- Social awareness, social cognition, social communication and social motivation subscales from SRS-2.
- Executive functions from Spanish adaptation of the Behavior Rating Inventory of Executive Function, Second Edition (BRIEF-2) (Gioia et al., 2017): This test of 63 items provides a total score known as the Global Index of Executive Function (EF), as well as scores on specific indexes for behavioral, emotional, and cognitive regulation. Each index is composed of multiple subscales. T-scores were used, where scores between 60 and 64 indicate slight elevation, scores between 65 and 69 suggest potential clinical significance, and scores above 69 represent clinical levels.
- Child Health and Illness Profile (CHIP-CE): We used the adapted version for the Spanish population (Rajmil et al., 2004), which has adequate psychometric properties and is useful for measuring perceived health status in a child health survey (Estrada et al., 2010). This questionnaire assesses various aspects of physical, emotional, and social health.
- Bruni’s Sleep Disorders Scale for Children (SDSC) (Bruni et al., 1996): We used the Spanish version which has good reliability and is considered an adequate instrument for assessing sleep disturbances in school-age children and adolescents (Pagerols et al., 2023). It consists of 27 items, and a cut-off score of 39 is considered indicative of a global sleep disorder.

### Other Variables: Psychological Problems, Nutritional Status, Physiological Habits and Sociodemographic Data

Parents responded to the following tests and a trained dietitian collected anthropometry prior to the intervention.

- Child Behavior Checklist (CBCL 6–18) (Achenbach and Rescorla, 2001): The parents completed the CBCL at baseline to evaluate the psychological problems of the children. T-scores specific to the Spanish population were obtained for scales such as withdrawn/depression, anxiety/depression, somatic complaints, social problems, thinking problems, attention problems, rule-breaking behavior, and aggressive behavior. Additionally, total scores for psychological problems, externalizing problems, and internalizing problems were calculated. T-scores between 65 and 69 suggest subclinical significance, and scores above 69 indicate clinical levels.
- Physical Activity Questionnaire (PAQ-C) (Kowalski et al., 2004; Manchola-Gonzalez et al., 2017): This questionnaire is suitable for elementary school-aged children (approximately 8–14 years) who have recess as a regular part of their school days.
- Food Consumption Frequency Questionnaire (FCFQ) (Trinidad et al., 2008): Validated in the Spanish population, this semi-quantitative questionnaire contains 45 items that assess the frequency of food and beverage consumption per week or per month over the past year. Parents completed the FCFQ before and after the intervention. The Spanish Diet Quality Index (SDQI) (Norte-Navarro & Ortiz-Moncada, 2011) was then calculated. This index provides a score between 0 and 100 points. These scores were then classified into three levels: ≥ 80, healthy; 50–79, needs improvement; and ≤ 49, unhealthy.
- Anthropometric Measurements: Weight and height were measured to calculate body mass index (BMI) and BMI-for-age (BMI z-score) using the WHO guidelines for child growth assessment. Macro-SPSS syntax files provided by the WHO Anthro and Anthro Plus software were utilized for the calculations.
- Sociodemographic Family Data Questionnaire: This questionnaire includes various small questionnaires covering the child's medical history, pregnancy information, and socio-demographic data of the family unit. The parents completed this questionnaire at baseline.
- We collected the information on intelligence quotient of the children from *Wechsler Scales of Intelligence for pre-school* (WPPSI-IV, WISC-IV or WISC V) (Wechsler, 2015): Those tests provide specific scores for verbal comprehension, perceptual reasoning, working memory and the processing speed, allowing to estimate the global IQ.

### Statistical Analysis

The statistical analysis was conducted on an intention-to-treat (ITT) basis, regardless of adherence to the nutritional intervention. Sensitivity tests were also performed. The normality of the sample distribution was assessed using the Shapiro–Wilk test. Continuous variables were analyzed using either t-tests or Mann–Whitney U-tests, while non-continuous variables were analyzed using chi-square tests.

Inter-group analyses were conducted to compare the differences in scores between the interventions. For these analyses, t-tests and analysis of covariance (ANCOVA) were used, adjusting for the variables sex, age, diagnosis, pharmacological treatment, emotional problems, baseline scores, BMI z-score, and diet quality. Intra-group differences before and after the intervention were performed to follow temporal changes within a group. Paired t-tests or Wilcoxon signed-rank analysis were used for this purpose.

In cases where age was found to be a significant factor influencing the effect of the intervention, analyses were repeated by separating the sample into two age groups: younger children aged 5 to 9 years, and older children aged 10 to 14 years. In that case, the overall results for ASD and ADHD, as well as the analyses stratified by age group (younger and older) for both diagnoses and each intervention group, will be presented.

To estimate the effect size of the intervention, Cohen's d (*d*) and adjusted Cohen's d were also calculated for both inter-group and intra-group analyses. To categorize the effect size, 4 levels were established. Cohen's d around 0.2 means a small effect size, around 0.5 means a moderate effect size, around 0.8 means a large effect size, and more than 1 means a very large effect size of the studied intervention.

Non-parametric tests were used, when necessary, but data is reported as means ± SE to express the severity of symptomatology. The percentage of improvement by severity levels (categories of clinical ranges) for the BRIEF-2, Conners, SRS-2 and CPT tests, was calculated using Chi-square analyses. Improvement was graded differently for each test when the score moved from a clinical or elevated range/category to a lower or normal/ non-clinical range/category.

Given the several primary outcomes, after correcting for multiple comparisons, a p value of < 0.01 was established for statistical significance. Analyses were performed with the IBM SPSS 28 statistical software.

## Results

### Sample Characteristics

Table 1 presents the socio-demographic, clinical, and nutritional characteristics of the study participants at baseline, categorized by diagnosis and intervention group. The average age of the participants was 9.68 ± 2.61 years, with boys comprising 77.5% of the sample. Among the participants, 11 children (12.50%) were diagnosed with both ASD and ADHD. In terms of ADHD presentations, the most prevalent was the combined presentation (ADHD-C) with a 75%. Only two participants with ASD had intellectual disability and none with ADHD did. The results of the SDQI indicated that the majority of children in the study required an improvement in their dietary habits. No significant differences were observed in any baseline variables between the intervention groups.

**Table 1:** Baseline descriptive data by diagnosis and intervention assigned group

		Diagnosis					
		ASD			ADHD		
		Placebo	Probiotic	p	Placebo	Probiotic	p
		n = 21	n = 21		n = 18	n = 20	
Sex, Boyss n(%)		18(85.7)	17(81)	0.679	14(77.8)	13(65)	0.386
Age, Years		9.57 ± 3.14	9.71 ± 2.48	0.871	10.11 ± 2.30	9.35 ± 2.52	0.339
PELP n(%)	Low	1(4.8)	1(4.8)	0.950	1(5.6)	3(15)	0.602
	Medium	12(57.1)	11(52.4)		9(50)	8(40)	
	High	8(38.1)	9(42.9)		8(44.4)	9(45)	
Comorbidity ASD + ADHD n(%)		6(28.6)	5(23.81)	0.726	-	-	-
ADHD Presentation n(%)	Inattentive	2(9.5)	1(4.8)	0.740	5(27.8)	3(15)	0.323
	Hyperactive-Impulsive	0	0		1(5.6)	0	
	Combined	4(19)	3(14.3)		12(66.7)	85(17)	
IQ		108.82 ± 14.63	94.38 ± 22.60	0.083	99.88 ± 17.01	103.29 ± 15.84	0.554
Pharmacological treatment, Yes n(%)		10(47.6)	6(28.6)	0.204	5(27.8)	4(20)	0.573
Diet Quality (SDQI)		60.24 ± 5.23	59.35 ± 5.96	0.614	56.81 ± 5.99	59.33 ± 7.22	0.252
	Unhealthy n(%)	-	-	-	2(11)	1(5)	0.485
	Need to improve n(%)	21(100)	20(100)		16(88.9)	19(95)	
	Healthy n(%)	-	-		-	-	-
BMI		17.75 ± 3.89	19.26 ± 5.59	0.313	18.23 ± 2.79	18.67 ± 4.44	0.723
BMI z-score		0.15 ± 1.50	0.66 ± 1.68	0.304	0.55 ± 1.38	0.67 ± 1.38	0.789
	Underweight n(%)	5(23.8)	6(28.6)	0.429	1(5.6)	2(10)	0.623
	Normal n(%)	10(47.6)	6(28.6)		9(50)	12(60)	
	OW and OB n(%)	6(28.6)	9(42.9)		8(44.4)	6(30)	
Physical Activity, score		2.27 ± 0.92	2.50 ± 0.69	0.402	2.83 ± 0.75	2.84 ± 0.93	0.961
CBCL	Anxious/depressed	62.35 ± 1.66	64.53 ± 1.80	0.422	62.94 ± 2.02	64.30 ± 1.88	0.650
	Depressed	65.95 ± 2.20	68.63 ± 2.55	0.446	58.44 ± 1.70	62.05 ± 1.90	0.229
	Somatic complaints	60.15 ± 1.69	63.00 ± 1.94	0.300	60.67 ± 2.28	58.80 ± 1.62	0.565
	Social problems	65.35 ± 1.81	64.89 ± 1.97	0.821	64.44 ± 2.58	62.30 ± 1.45	0.838
	Thought problems	64.75 ± 1.92	63.47 ± 2.05	0.714	59.22 ± 1.90	62.00 ± 1.95	0.403
	Attention problems	65.60 ± 1.68	68.79 ± 3.31	0.821	70.72 ± 2.31	69.30 ± 2.06	0.587
	Rule-breaking behavior	54.75 ± 1.14	55.05 ± 1.30	0.819	56.83 ± 1.52	61.90 ± 1.92	0.093
	Aggressive behavior	59.65 ± 1.76	62.89 ± 2.45	0.430	64.44 ± 2.78	64.60 ± 1.91	0.872
	Internalizing problems	64.15 ± 1.68	67.63 ± 1.61	0.259	62.78 ± 1.71	63.25 ± 1.87	0.539
	Externalizing problems	57.00 ± 1.94	56.47 ± 3.37	0.987	60.80 ± 2.87	61.53 ± 2.48	0.791
	Total Score	64.15 ± 1.56	64.13 ± 2.23	0.854	64.54 ± 2.44	64.94 ± 1.66	0.801
Primary endpoints							
Conners' test	Inattention score	75.76 ± 3.24	69.84 ± 3.50	0.195	80.50 ± 2.36	77.05 ± 2.31	0.296
	Hyperactivity-Impulsivity score	69.29 ± 3.53	70.16 ± 4.52	0.796	69.83 ± 3.90	77.20 ± 3.23	0.184
SRS-2	Restricted interests and repetitive behaviors	74.29 ± 2.23	71.70 ± 2.60	0.441	-	-	-
	Social Communication and Interaction	71.29 ± 2.43	70.80 ± 2.48	0.764	-	-	-
	Total score	72.48 ± 2.41	72.10 ± 2.45	0.896	-	-	-
CPT test	Detectability	56.88 ± 2.37	56.61 ± 1.83	0.667	57.61 ± 2.48	57.16 ± 1.80	0.637
	Omissions	61.29 ± 4.32	60.61 ± 3.42	0.804	63.89 ± 4.15	62.21 ± 4.09	0.831
	Commissions	53.47 ± 2.15	52.44 ± 2.23	0.716	54.39 ± 1.70	52.32 ± 1.89	0.474
	Perseverations	56.12 ± 3.67	57.61 ± 3.15	0.540	60.56 ± 4.34	57.68 ± 2.55	0.867
	Hit reaction time	53.59 ± 2.12	58.78 ± 2.22	0.113	56.94 ± 2.03	56.58 ± 2.51	0.927
Secondary endpoints							
Conners' test	Learning problems score	60.90 ± 2.62	60.16 ± 3.60	0.569	64.06 ± 3.59	62.10 ± 1.86	0.704
	Executive functioning score	65.71 ± 3.25	64.32 ± 3.11	0.684	69.39 ± 2.88	69.05 ± 2.37	0.977
	Defiance/Aggression score	55.24 ± 2.56	53.74 ± 2.63	0.436	62.00 ± 3.75	60.35 ± 3.54	0.638
	Peer Relations	79.86 ± 3.08	81.89 ± 2.59	0.976	64.67 ± 4.06	65.00 ± 4.16	0.930
SRS-2	Social awareness	65.24 ± 2.28	63.75 ± 2.38	0.895	-	-	-
	Social cognition	70.33 ± 2.45	71.95 ± 2.57	0.744	-	-	-
	Social communication	69.67 ± 2.76	69.35 ± 2.79	0.845	-	-	-
	Social motivation	68.29 ± 2.60	67.05 ± 2.47	0.774	-	-	-
Brief test	Behavioral regulation index score	64.62 ± 2.10	61.62 ± 2.92	0.274	65.67 ± 3.77	66.30 ± 2.17	0.884
	Emotional regulation index score	69.48 ± 2.22	68.29 ± 3.66	0.743	67.22 ± 3.03	63.10 ± 3.08	0.342
	Cognitive regulation index score	67.79 ± 2.98	65.71 ± 2.67	0.542	72.67 ± 3.15	68.00 ± 2.22	0.225
	Global index of executive functioning score	70.58 ± 2.02	67.95 ± 3.04	0.222	73.39 ± 3.22	69.15 ± 2.10	0.364
CHIP-CE	Satisfaction score	36.65 ± 4.15	36.93 ± 2.64	0.554	34.69 ± 3.33	33.82 ± 2.58	0.629
	Comfort score	42.80 ± 2.57	40.74 ± 2.49	0.580	41.67 ± 2.80	41.73 ± 2.17	0.826
	Resilience score	37.96 ± 4.13	35.04 ± 2.88	0.571	39.54 ± 2.73	37.53 ± 2.44	0.365
	Risk avoidance score	41.26 ± 2.88	39.35 ± 3.20	0.753	39.05 ± 3.10	32.86 ± 3.50	0.266
	Achievement score	32.98 ± 2.78	34.14 ± 3.35	0.706	34.53 ± 2.96	35.79 ± 1.83	0.781
Bruni's test	Global sleep disorder score	14.00 ± 1.62	16.19 ± 2.92	0.772	16.28 ± 2.54	14.05 ± 1.89	0.736
	Global sleep disorder, Yes n(%)	0	2(9.5)	0.157	1(5.3)	0	0.299

Analyses conducted on an intention-to-treat-basis. T-test or Mann Whitney’s U (for non-normally variables) were used for quantitative variables and Chi^2^ for qualitative. Quantitative variables are expressed in mean ± SD, primary and secondary endpoints scores are expressed in mean ± SE. PELP, parents’ educational level and profession; IQ, intelligence quotient; ADHD, attention deficit/hyperactivity disorder; SDQI, Spanish diet quality index; BMI, body mass index; OW and OB, overweight and obesity; GI, gastrointestinal; CBCL, Child Behavior Checklist; CPT, Continuous performance test. Significant differences p < 0.01. *Including children with both diagnoses

Two children did not complete the minimum dosage of the study product (≥ 75%) with 54% and 73% of the intakes. Of the participants, the 82.5% took more than 94% of the product (from 85 to 90 of the individual doses).

### Primary Endpoints

For the Conners test subscales (Table 2), inter-group analyses did not yield any significant findings. Nevertheless, ANCOVA analyses indicated that age is a factor that may affect the probiotic effect on the hyperactivity-impulsivity symptoms. Accordingly, analyses stratified by diagnosis and age and adjusted for the various covariates showed that the probiotic intervention in younger children with ASD improved hyperactivity-impulsivity symptoms (p = 0.036), with a very large effect size (*d* = 1.245). On the other side, in younger children with ADHD, although no statistical trends were observed, the Cohen’s d showed a moderate-to-large size effect (0.692) in favor of the probiotic-treated group. The intra-group analysis within the probiotic group, revealed an improving trend in hyperactivity-impulsivity scores in children with ASD (Mean ± SE: score decreased from 70.2 ± 4.5 to 64.8 ± 3.8, p = 0.017), with moderate size effect (*d* = 0.537). When the intra-group analysis was made by age, the younger children with ASD showed a decrease in hyperactivity-impulsivity symptoms with an effect size between moderate to large (*d* = −0.606). For ADHD, only the younger children treated with probiotics displayed a meaningful trend toward decreased hyperactivity-impulsivity (Mean ± SE: score decreased from 80 ± 3.5 to 69.3 ± 2.7, p = 0.016) with a very large size effect (*d* = −1.030).

**Table 2:** Primary endpoints: scores of the Conners, SRS-2 and CPT tests by intervention group

		Placebo Group		Probiotic Group					
		Mean ± SE	Median [IQR]	Mean ± SE	Median [IQR]	Cohen’s d	*P* value	Cohen’s d Adjusted	*P* Value Adjusted
Conners' test									
Inattention score									
ASD	Baseline	75.76 ± 3.24	76[23]	69.84 ± 3.51	74[33]				
	12 week	75.19 ± 3.08	79[28]	68.74 ± 3.91	71[33]				
	Score change	–0.57 ± 2.55	0[8]	–1.11 ± 2.30	0[6]	0.049	0.878	0.261	0.493
ADHD	Baseline	80.50 ± 2.36	85[16]	77.05 ± 2.31	76[17]				
	12 week	76.89 ± 2.76	77[21]	74.65 ± 2.36	75[17]				
	Score change	–3.61 ± 1.92	–3[11]	–2.40 ± 2.33	2[15]	–0.129	0.694	–0.004	0.990
Hyperactivity-Impulsivity score									
ASD	Baseline	69.29 ± 3.53	69[31]	70.16 ± 4.52	79[42]				
	12 week	65.19 ± 3.53	65[31]	64.79 ± 3.80	71[34]				
	Score change	–4.10 ± 1.98	–3[11]	*–5.37 ± 2.29*	*–4[11]*	0.133	0.676	0.006	0.987
Young ASD	Score change	–3.27 ± 2.79	–5[10]	–7.20 ± 3.76	–2.5[13]	0.371	0.406	1.245	0.036
Older ASD	Score change	–5.00 ± 2.96	–2[13]	–3.33 ± 2.52	–4[8]	–0.195	0.677	–0.610	0.356
ADHD	Baseline	69.83 ± 3.90	71[28]	77.20 ± 3.23	82[22]				
	12 week	68.22 ± 3.80	68[30]	73.00 ± 3.51	75[29]				
	Score change	–1.61 ± 2.42	0[10]	–4.20 ± 2.51	–0.5[20]	0.240	0.464	0.225	0.535
Young ADHD	Score change	–3.00 ± 4.59	3[19]	*–10.70 ± 3.29*	*–10[16]*	0.692	0.181	0.316	0.601
Older ADHD	Score change	–0.73 ± 2.83	0[9]	2.30 ± 2.53	2[8]	–0.346	0.438	0.115	0.834
SRS test †									
Restricted interests and repetitive behaviors score									
	Baseline	74.29 ± 2.23	73[14]	71.70 ± 2.60	70[17]				
	12 week	71.48 ± 2.69	69[19]	71.20 ± 2.82	73[20]				
	Score change	–2.81 ± 1.58	–4[9]	–0.50 ± 1.51	–0.5[13]	–0.439	0.168	–0.697	0.056
Social Communication and Interaction									
	Baseline	71.29 ± 2.43	71[16]	70.80 ± 2.48	68[17]				
	12 week	68.76 ± 2.42	68[18]	69.75 ± 2.52	72[17]				
	Score change	–2.52 ± 1.46	–3[8]	–1.05 ± 1.32	–1[10]	–0.234	0.459	–0.699	0.056
Total score									
	Baseline	72.48 ± 2.42	74[15]	72.10 ± 2.47	71[19]				
	12 week	70.05 ± 2.47	69[19]	70.75 ± 2.45	70[17]				
	Score change	–2.43 ± 1.43	–3[9]	–1.35 ± 1.54	–3[10]	–0.257	0.415	–0.909	0.015
CPT test									
Detectability score									
ASD	Baseline	56.88 ± 2.38	53[14]	56.53 ± 1.94	54[15]				
	12 week	52.18 ± 2.41	59[17]	53.41 ± 2.51	58[11]				
	Score change	*–4.71 ± 1.86*	*–4[6]*	–3.12 ± 1.57	–3[10]	–0.224	0.519	–0.086	0.821
ADHD	Baseline	57.61 ± 2.48	58[21]	57.16 ± 1.80	56[11]				
	12 week	56.33 ± 2.61	59[15]	54.79 ± 1.98	59[14]				
	Score change	–1.28 ± 1.87	0[8]	–2.37 ± 1.40	–1.5[8]	0.155	0.641	0.225	0.525
Omissions score									
ASD	Baseline	61.29 ± 4.32	49[18]	58.88 ± 3.13	50[23]				
	12 week	54.59 ± 3.01	52[31]	58.18 ± 3.69	60[19]				
	Score change	–6.71 ± 3.35	–1[7]	–0.71 ± 3.13	–2[15]	–0.449	0.200	–0.208	0.590
ADHD	Baseline	63.89 ± 4.15	55[24]	62.21 ± 4.09	57[24]				
	12 week	60.39 ± 3.82	58[34]	60.74 ± 3.75	56[33]				
	Score change	–3.50 ± 3.85	–0.5[17]	–1.47 ± 3.52	–0.5[18]	–0.128	0.700	0.134	0.706
Commissions score									
ASD	Baseline	53.47 ± 2.15	46[12]	52.94 ± 2.31	46[11]				
	12 week	48.53 ± 2.15	54[16]	46.88 ± 2.00	54[13]				
	Score change	*–4.94 ± 1.66*	*–5[12]*	–6.06 ± 1.21*	–6[7]*	0.187	0.590	0.089	0.815
ADHD	Baseline	54.39 ± 1.70	51[14]	52.32 ± 1.89	51[16]				
	12 week	52.11 ± 2.55	55[12]	50.47 ± 2.07	53[8]				
	Score change	–2.28 ± 1.96	–1[16]	–1.84 ± 1.63	–2[8]	–0.056	0.865	0.031	0.930

Analyses conducted on an intention-to-treat-basis. Mean ± SE. * *p* < 0.01, statistical trend highlighted in italics, intra-group difference by Paired *t*-test analysis or Wilcoxon signed-ranks test (non-normally distributed variables). Between groups analysis by *T*-test or Mann Whitney U (non-normally distributed variables). Inter-groups difference analysis by *T*-test. ANCOVA was used for Inter-group adjusted analysis by sex, age, pharmacological treatment, diagnosis, emotional problems, baseline scores, zBMI, and the quality of the diet

In relation to autism test scores (SRS-2), probiotics showed no beneficial effect in the inter-group analyses (Table 2). On the contrary, an improvement trend in total score was found for the placebo group (p = 0.015, with a large size effect: *d* = −0.909). In the intra-group analyses, the probiotic intervention also did not exert any significant effects on autism symptoms (SRS-2) when compared to placebo. Regarding the data of the CPT (Table 2), non-significant findings were showed by the inter-group analyses. The intra-group analyses showed a significant decrease in commission errors (p = 0.001) with a very large size effect (*d* = −1.216) in the children with ASD treated with probiotic. However, in the placebo-treated group a trend was also found (p = 0.013, *d* = −0.721). Regarding detectability, a trend of improvement in the placebo group (p = 0.016, *d* = −0.612) was found in children with ASD. For the probiotic group of children with ASD, only a moderate effect size was demonstrated (*d* = −0.482).

### Secondary Endpoints

No significant differences were found at baseline and after intervention between the placebo group and the probiotic group in any of the diagnoses.

Supplementary Table 1 presents data of the secondary outcomes of the Conners and SRS-2 subscales and the BRIEF-2, CHIP-CE and Bruni test scores. No significant results were shown by the inter-group analyses. Only in the CHIP-CE, the probiotic group showed intra-group improvements in scores on the comfort subscale [Mean ± SE (score from 40.7 ± 2.5 to 46.2 ± 2.5, p = 0.010)] with a large size effect (*d* = 0.722) in children with ASD.

According to the Bruni scores, the intervention showed no effect on sleep.

In terms of the percentage of improvement by severity levels after the intervention, as measured by the BRIEF-2, Conners, SRS-2, and CPT tests—using validated cut-off scores—only the Social Awareness scale of the SRS-2 showed clinically an improvement trend. Specifically, 42.1% (n = 8) of participants who showed improvement were in the probiotic group, with 62.5% (n = 5) of them moving from clinical ranges to the 'normal' range. On the other hand, the 11.1% (n = 2) of the participants of the placebo group showed improvement, and both participants moved from clinical ranges to the 'normal' range (p = 0.034).

Outside the secondary endpoints, we analyzed whether dietary changes occurred. In this regard, there were no differences in food consumption as a result of the intervention.

## Discussion

This study represents a contribution to the limited body of research examining the efficacy of dietary supplements, particularly probiotics, in ameliorating symptomatology in children diagnosed with ASD and/or ADHD. The results indicated a slight improvement in hyperactive-impulsive symptoms among younger children, as measured by behavioral assessments. The children recruited for this study mainly came from higher socioeconomic backgrounds (more than 90% belonged to the upper-middle class) and they did not present severe behavioral, emotional, or sleep problems. Furthermore, the sample did not include children with severe symptomatology or with intellectual disability, which decreases the chances of significant improvement (except in two children with ASD).

The primary objective of this randomized, double-blind, placebo-controlled trial was to investigate the potential positive effects of a probiotic mixture containing *Lactiplantibacillus plantarum* and *Levilactobacillus brevis* on the severity of symptomatology associated with ASD and ADHD. The rationale for this investigation stems from prior studies suggesting that some probiotic strains from said species exhibit the capacity to produce GABA and dopamine (Barrett et al., 2012; Cheng et al., 2019; Dash et al., 2022), the dysregulation of which has been implicated in the pathophysiology of both ASD and ADHD (Mishra et al., 2022; Port et al., 2017; Purkayastha et al., 2015; Puts et al., 2020; Shirvani-Rad et al., 2022).

The probiotic intervention did not significantly affect core ASD symptoms, including social interaction difficulties, communication problems, and repetitive restrictive behaviors, as reflected by the SRS-2 mean scores. This is consistent with findings from other studies, which similarly reported no improvement in core ASD symptoms (Barba-Vila et al., 2024; Lee et al., 2024; Liu et al., 2019). In contrast, using a combination of *Lactobacillus* and Bifidobacteria, Shaaban et al., (2017) did find a significant improvement in the severity of autism. Parracho et al. (2010) also utilized *L. plantarum* in their study, and despite having a smaller sample size, they observed behavioral improvements using alternative assessment tools such as the Development Behavior Checklist (DBC). On the other hand, the systematic review conducted by He et al. (2023) reported that improvements in behavior were observed only in studies involving the combination of probiotic strains with other substances, such as a mixture of different probiotic strains (Arnold et al., 2019; Li et al., 2021; Santocchi et al., 2020), fructo-oligosaccharides (FOS) (Y. Wang et al., 2020), or a bovine colostrum product (BCP) (Sanctuary et al., 2019).

In our study and contrary to our hypothesis, not only did the probiotics not improve the symptoms of autism, but the improvement in the SRS-2’s Total score was shown in the placebo group, a result that is challenging to explain. We could not detect variables that differentiated this group from the probiotic group concerning severity level, time of year in which the intervention took place, diet changes or modifications in concurrent treatments that could explain these findings. Nevertheless, an improvement in the severity levels of social awareness was observed in the probiotic group (from symptomatic to normal category), consistent with data with boys with ASD from the study of Liu et al. (2019) although there was no significant impact on scores.

Inattention, a core symptom of ADHD, was not significantly affected by the probiotic intervention according to the attention subscale of the Conners test nor through the data of the neuropsychological test CPT applied to the children. However, intra-group analyses on this test, showed significant improvements and a very large size effect of the intervention in children with autism on the Commission scores, which is related to impulsivity and inhibitory control. It agrees with the changes observed in hyperactivity-impulsivity, as perceived behaviorally by parents (Conners’s test), found in young children with autism who received probiotics. Our results are in partial agreement with those of Wu et al., (2021) which utilizing *L. plantarum* as an intervention in individuals with Tourette’s syndrome reported improvement in the detectability and commission scores of the CPT test, despite showing no positive results in tic symptomatology (Wu et al., 2021). In relation to hyperactivity/impulsivity, our findings indicate that the probiotic intervention has an impact on this symptomatology among younger children (aged 5 to 9 years), diagnosed with ASD and or ADHD, as evidenced by the scores obtained from the Conners test. This effect in both disorders may be related to the presence of shared symptomatology between ADHD and ASD, and to common neurophysiological bases (Dellapiazza et al., 2021; McClain et al., 2017; Rau et al., 2020). The age of the participants played an important role in the observed effects. A very large effect size was shown in younger children with ASD and a moderate to large effect size in those with ADHD. These findings were consistent with the intra-group analyses, which showed a statistical trend of improvement and a great size effect after the probiotic intervention, in this younger age group and for both diagnoses. Hyperactive-impulsive symptoms are more frequent in younger children and decrease with the age (Biederman et al., 2000; Canals-Sans et al., 2021; Ramelli et al., 2010), which support that the effect of intervention was more evident in this group of age. Although Wang’s study (2022) was a single-armed study and used other assessment tools and probiotic species (*B. bifidum*), an improvement in hyperactivity/impulsivity symptoms was also observed after the probiotic intervention. Our findings also corroborate other previous research like a probiotic intervention with *L. plantarum* in children diagnosed with ASD, which reported an improvement in hyperactivity in younger children, although their sample was made up exclusively of boys (Liu et al., 2019). On the other hand, an association was found between hyperactivity symptoms and lower alpha diversity of the gut microbiota in children with ADHD (Prehn-Kristensen et al., 2018). These authors did not find that association with the inattention symptoms. The results obtained in our study align with those findings, as we observed improvements specifically in hyperactivity. We did not report results on gut microbiota composition; thus, our observation refers only to clinical data and not to correlations with gut microbiota. However, it is worth noting that another study reported contrasting results, indicating a correlation between microbiota diversity and inattention symptoms. The participants in that study were older, ranging from 13 to 29 years (Szopinska-Tokov et al., 2020), a stage at which inattention symptoms tend to be higher than hyperactivity symptoms due to the evolving nature of the disorder itself (Biederman et al., 2000).

A recent systematic review concludes that probiotics represent a viable adjunctive treatment for ADHD. However, it is pertinent to observe that in the majority of studies, the intervention was not only composed by one or more probiotic strains, with some trials incorporating methylphenidate alongside, which could be the reason of the symptomatology improvement (Nahidi et al., 2024).

The lack of significant improvement in the core symptoms of ASD and ADHD could be attributed to the chronic nature of these disorders, which typically develop early in life. Some studies suggest that probiotic interventions may be more effective when administered during early developmental stages, such as infancy or even gestation, potentially reducing the risk of developing ASD or ADHD (Pärtty et al., 2015; Slykerman et al., 2018). Future research might explore the timing of probiotic interventions to better understand their potential in preventing or mitigating symptomatology before these disorders fully manifest. However, we hypothesized an improvement of core and/or other symptoms related to different areas of functioning, which has been discretely verified.

The quality of life for children with ASD and ADHD can vary widely based on factors such as condition severity, individual characteristics, support systems, and interventions. Generally, the sample in this study had a moderate level of well-being without severe issues. The inter-group analysis of the CHIP-CE test did not show significant differences between groups, and the effect size of the probiotic intervention was small. However, intra-group analyses revealed improvements in comfort levels with a large size effect among children with ASD who received probiotics, suggesting that even when broad changes in quality of life are not apparent, specific areas could benefit from probiotic treatment. Another study on children with ASD aged 3 to 12, using a different probiotic formulation and assessed with PedsQL, found no significant quality of life improvements attributable to the probiotics, as both intervention groups showed similar changes (Arnold et al., 2019). Conversely, a study on children with ADHD self-reported physical, emotional, and social improvements, and better school functioning according to PedsQL, though these improvements were not observed by parents or teachers (Kumperscak et al., 2020).

### Strengths and Limitations

This study adds to the growing body of research on the potential role of probiotics as adjunctive treatments for ASD and ADHD, with several key strengths. The randomized, double-blind design minimizes selection bias and ensures robust comparability between groups, while the placebo control enables a clear assessment of probiotic-specific effects. The use of validated, high-reliability outcome measures and standardized scoring systems enhances the study's rigor and allows for direct comparisons with previous research. Additionally, the combined use of both behavioral assessments, based on parental/guardian reports, and neuropsychological measures provides a more comprehensive view of the intervention’s effects, enhancing sensitivity in detecting subtle changes.

However, several limitations must be acknowledged. The relatively small sample size reduces the study's statistical power and limits the generalizability of the findings. A larger sample could enable more detailed subgroup analyses based on age, clinical characteristics, ADHD presentations, ASD severity, comorbidities, and cognitive levels. For this reason, moderation analyses to explore age-specific effects could not been performed. The relatively low symptom severity in the sample may have limited the scope for significant improvement, contributing to the modest effect sizes observed. Moreover, while the probiotic strains used have shown promise in neurodevelopmental research due to their roles in neurotransmitter production, the intervention was not tailored to participants’ individual baseline microbiota. A more personalized approach could have yielded more robust results. Lastly, the 12-week intervention period may not have been long enough to capture more profound or long-term effects, suggesting that extended treatment durations should be considered in future studies.

## Conclusions

Overall, this study suggests that probiotics may offer targeted benefits, particularly in alleviating hyperactivity-impulsivity symptoms in younger children with ASD and/or ADHD. However, no significant improvements were observed in the core symptoms of ASD. These findings highlight the potential for probiotics as a complementary treatment in specific neurodevelopmental domains, but further research is essential to better understand the underlying mechanisms and to optimize the use of probiotics.

## Supplementary Information

Below is the link to the electronic supplementary material.

Supplementary file1 (DOCX 49 KB)

## Data Availability

The datasets generated and/or analyzed during the current study are not publicly available due to privacy, but are available from the corresponding author on reasonable request.
