# Supplementary material for: Effect of Probiotics on the Symptomatology of Autism Spectrum Disorder and/or Attention Deficit/Hyperactivity Disorder in Children and Adolescents: Pilot Study
Source: Res Child Adolesc Psychopathol. 2025 Jan 11;53(2):163–78. doi: 10.1007/s10802-024-01278-7 (PMC11845535; doi:10.1007/s10802-024-01278-7)
Supplement: Supplementary file 1 — Supplementary file1 (DOCX 49 KB) [file 10802_2024_1278_MOESM1_ESM.docx]

| **Supplementary Table 1. Secondary endpoints: scores of the Conners’, SRS, BRIEF, CHIP and BRUNI test** | | | | | | | | | | | | |
| --- | --- | --- | --- | --- | --- | --- | --- | --- | --- | --- | --- | --- |
|  |  | **Placebo Group** | | | **Probiotic Group** | | **Cohen’s d** | **P value** | **Cohen’s d Adjusted** | | **P Value Adjusted** | |
|  |  | Mean±SE | | Median [IQR] | Mean±SE | Median [IQR] |  |  |  | |  | |
| **Conners' test** | |  | |  |  |  |  |  |  | |  | |
| **Learning problems score** | | | |  |  |  |  |  |  | |  | |
| ASD | Baseline | 60.90±2.62 | | 60[17] | 60.16±3.60 | 56[29] |  |  |  | |  | |
|  | 12 week | 63.05±2.89 | | 61[21] | 62.37±3.82 | 61[29] |  |  |  | |  | |
|  | Score change | 2.14±2.13 | | 0[18] | 2.21±2.10 | 0[10] | -0.007 | 0.982 | 0.128 | | 0.723 | |
| ADHD | Baseline | 64.06±3.59 | | 67[26] | 62.10±1.86 | 62[11] |  |  |  | |  | |
|  | 12 week | 61.78±3.42 | | 60[19] | 61.30±3.03 | 58[17] |  |  |  | |  | |
|  | Score change | -2.28±1.93 | | 0[11] | -0.80±2.13 | 0[9] | -0.166 | 0.613 | -0.348 | | 0.328 | |
| **Executive functioning score** | | | |  |  |  |  |  |  | |  | |
| ASD | Baseline | 65.71±3.25 | | 65[26] | 64.32±3.11 | 61[19] |  |  |  | |  | |
|  | 12 week | 63.86±3.05 | | 63[20] | 62.84±3.12 | 61[18] |  |  |  | |  | |
|  | Score change | -1.86±2.29 | | 0[16] | -1.47±2.05 | -2[15] | -0.039 | 0.902 | -0.113 | | 0.755 | |
| ADHD | Baseline | 69.39±2.88 | | 69[21] | 69.05±2.37 | 69[15] |  |  |  | |  | |
|  | 12 week | 69.06±2.52 | | 69[17] | 70.10±2.73 | 70[15] |  |  |  | |  | |
|  | Score change | -0.33±2.63 | | -2[18] | 1.05±2.26 | 0[15] | -0.130 | 0.691 | -0.149 | | 0.669 | |
| **Defiance/Aggression score** | | | |  |  |  |  |  |  | |  | |
| ASD | Baseline | 55.24±2.56 | | 51[17] | 53.74±2.63 | 51[14] |  |  |  | |  | |
|  | 12 week | 51.95±2.34 | | 46[12] | 55.37±3.18 | 51[12] |  |  |  | |  | |
|  | Score change | -3.29±3.13 | | 0[6] | 1.63±1.54 | 0[7] | -0.432 | 0.181 | -0.250 | | 0.487 | |
| ADHD | Baseline | 62.00±3.75 | | 59[25] | 60.35±3.54 | 58[24] |  |  |  | |  | |
|  | 12 week | 56.94±3.32 | | 53[20] | 62.20±3.60 | 60[31] |  |  |  | |  | |
|  | Score change | -5.06±3.25 | | 0[17] | 1.85±3.38 | 0[12] | -0.476 | 0.152 | -0.739 | | 0.041 | |
| **Peer Relations** | | |  |  |  |  |  |  |  | |  |  |
| ASD | Baseline | | 79.86±3.08 | 90[2] | 81.89±2.59 | 90[19] |  |  |  | |  |  |
|  | 12 week | | 83.81±2.82 | 90[4] | 82.80±2.50 | 90[15] |  |  |  | |  |  |
|  | Score change | | 3.95±2.47 | 0[6] | 0.89±1.89 | 0[2] | 0.307 | 0.339 | 0.120 | | 0.741 |  |
| ADHD | Baseline | | 64.67±4.06 | 60[32] | 65.00±4.16 | 61[39] |  |  |  | |  |  |
|  | 12 week | | 64.50±3.85 | 65[28] | 68.55±4.06 | 68[37] |  |  |  | |  |  |
|  | Score change | | -0.17±2.80 | 0[15] | 3.55±3.18 | 0[9] | -0.282 | 0.390 | -0.227 | | 0.514 |  |
| **SRS test †** | |  | |  |  |  |  |  |  | |  | |
| **Social awareness score** | |  | |  |  |  |  |  |  | |  | |
|  | Baseline | 65.24±2.28 | | 64[15] | 63.75±2.38 | 62[10] |  |  |  | |  | |
|  | 12 week | 63.62±2.55 | | 64[21] | 62.60±2.60 | 62[19] |  |  |  | |  | |
|  | Score change | -1.62±1.56 | | 0[10] | -1.15±2.17 | 0[15] | -0.050 | 0.875 | -0.079 | | 0.824 | |
| **Social cognition score** | |  | |  |  |  |  |  |  | |  | |
|  | Baseline | 70.33±2.45 | | 72[14] | 71.95±2.57 | 73[19] |  |  |  | |  | |
|  | 12 week | 68.76±2.54 | | 68[16] | 70.90±2.16 | 74[12] |  |  |  | |  | |
|  | Score change | -1.57±1.42 | | -3[10] | -1.05±2.03 | 0[14] | -0.139 | 0.659 | -0.626 | | 0.091 | |
| **Social communication score** | | | |  |  |  |  |  |  | |  | |
|  | Baseline | 69.67±2.76 | | 71[19] | 69.35±2.79 | 69[21] |  |  |  | |  | |
|  | 12 week | 67.86±2.88 | | 64[25] | 67.75±2.67 | 68[16] |  |  |  | |  | |
|  | Score change | -1.81±2.11 | | 0[15] | -1.60±1.20 | -2[10] | -0.128 | 0.682 | -0.638 | | 0.080 | |
| **Social motivation score** | |  | |  |  |  |  |  |  | |  | |
|  | Baseline | 68.29±2.60 | | 64[19] | 67.05±2.47 | 65[19] |  |  |  | |  | |
|  | 12 week | 65.29±2.59 | | 64[15] | 67.45±2.88 | 68[25] |  |  |  | |  | |
|  | Score change | -3.00±1.79 | | -2[7] | 0.40±2.16 | 0[13] | -0.431 | 0.175 | -0.708 | | 0.053 | |
| **Brief test** |  |  | | |  | |  |  |  | |  | |
| **Behavioral regulation index score** | | | |  |  |  |  |  |  | |  | |
| ASD | Baseline | 64.62±2.10 | | 69[8] | 61.50±3.06 | 68[28] |  |  |  | |  | |
|  | 12 week | 64.81±2.32 | | 66[18] | 60.75±3.57 | 59[16] |  |  |  | |  | |
|  | Score change | 0.19±1.30 | | 0[10] | -0.75±1.78 | -2[7] | 0.134 | 0.760 | 0.040 | | 0.909 | |
| ADHD | Baseline | 65.67±3.77 | | 66[11] | 66.30±2.17 | 60[23] |  |  |  | |  | |
|  | 12 week | 63.11±3.18 | | 64[18] | 66.50±2.36 | 68[19] |  |  |  | |  | |
|  | Score change | -2.56±2.44 | | -2[18] | 0.20±1.76 | 1[11] | -0.302 | 0.358 | -0.638 | | 0.074 | |
| **Emotional regulation index score** | | | |  |  |  |  |  |  | |  | |
| ASD | Baseline | 69.48±2.22 | | 72[10] | 68.05±3.84 | 67[22] |  |  |  | |  | |
|  | 12 week | 68.71±2.05 | | 67[16] | 68.05±3.93 | 67[21] |  |  |  | |  | |
|  | Score change | -0.76±1.67 | | -2[10] | 0±2.25 | 0[12] | -0.086 | 0.786 | -0.113 | | 0.749 | |
| ADHD | Baseline | 67.22±3.03 | | 69[17] | 63.10±3.09 | 69[15] |  |  |  | |  | |
|  | 12 week | 64.72±2.73 | | 73[19] | 61.30±3.11 | 68[12] |  |  |  | |  | |
|  | Score change | -2.50±2.28 | | -3[13] | -1.80±2.37 | 0[12] | -0.069 | 0.833 | -0.092 | | 0.792 | |
| **Cognitive regulation index score** | | | |  |  |  |  |  |  | |  | |
| ASD | Baseline | 67.79±2.98 | | 71[14] | 65.65±2.80 | 67[30] |  |  |  | |  | |
|  | 12 week | 67.58±2.88 | | 65[14] | 66.25±3.21 | 61[20] |  |  |  | |  | |
|  | Score change | -0.21±1.37 | | 0[7] | 0.6±1.45 | 0[10] | -0.130 | 0.687 | -0.322 | | 0.379 | |
| ADHD | Baseline | 72.67±3.15 | | 66[21] | 68.00±2.22 | 64[20] |  |  |  | |  | |
|  | 12 week | 71.4±2.9 | | 66[28] | 68.00±2.11 | 66[5] |  |  |  | |  | |
|  | Score change | -1.28±1.85 | | -0.5[13] | 0±1.76 | -2[12] | -0.163 | 0.619 | -0.033 | | 0.928 | |
| **Global index of executive functioning score** | | | | |  |  |  |  |  | |  | |
| ASD | Baseline | 70.58±2.02 | | 68[10] | 67.80±3.19 | 66[21] |  |  |  | |  | |
|  | 12 week | 70.00±2.42 | | 68[15] | 67.70±3.62 | 67[20] |  |  |  | |  | |
|  | Score change | -0.58±1.33 | | 0[10] | -0.10±1.60 | 0.5[7] | -0.073 | 0.821 | -0.245 | | 0.500 | |
| ADHD | Baseline | 73.39±3.22 | | 72[20] | 69.15±2.10 | 67[14] |  |  |  | |  | |
|  | 12 week | 70.94±2.88 | | 72[20] | 68.50±2.06 | 68[12] |  |  |  | |  | |
|  | Score change | -2.44±1.90 | | 0[14] | -0.65±1.85 | -2[11] | -0.220 | 0.503 | -0.235 | | 0.505 | |
| **CHIP test** | |  | | |  | |  |  | |  | | |
| **Satisfaction score** | |  | |  |  |  |  |  |  | |  | |
| ASD | Baseline | 35.65±4.15 | | 36[24] | 36.93±2.64 | 40[15] |  |  |  | |  | |
|  | 12 week | 38.27±4.05 | | 35[25] | 39.27±2.98 | 38[19] |  |  |  | |  | |
|  | Score change | 2.63±1.48 | | 3[9] | 2.34±2.61 | 0[16] | 0.030 | 0.924 | -0.102 | | 0.778 | |
| ADHD | Baseline | 34.69±3.33 | | 38[22] | 33.82±2.58 | 37[17] |  |  |  | |  | |
|  | 12 week | 37.30±3.82 | | 38[17] | 37.74±2.48 | 34[14] |  |  |  | |  | |
|  | Score change | 2.61±2.78 | | 0[15] | 3.92±2.95 | 0[16] | -0.104 | 0.750 | -0.013 | | 0.970 | |
| **Comfort score** | |  | |  |  |  |  |  |  | |  | |
| ASD | Baseline | 42.80±2.57 | | 46[8] | 40.74±2.49 | 48[15] |  |  |  | |  | |
|  | 12 week | 48.42±1.59 | | 45[16] | 46.23±2.51 | 43[19] |  |  |  | |  | |
|  | Score change | 5.62±2.69 | | 2[16] | *5.49±1.80* | *2[11]* | 0.012 | 0.968 | 0.210 | | 0.628 | |
| ADHD | Baseline | 41.67±2.80 | | 46[9] | 41.73±2.17 | 46[11] |  |  |  | |  | |
|  | 12 week | 46.48±1.76 | | 44[11] | 44.44±2.22 | 42[16] |  |  |  | |  | |
|  | Score change | 4.81±2.66 | | 4[11] | 2.70±1.37 | 2[8] | 0.235 | 0.474 | 0.239 | | 0.492 | |
| **Resilience score** | |  | |  |  |  |  |  |  | |  | |
| ASD | Baseline | 37.96±4.13 | | 39[14] | 35.04±2.88 | 38[13] |  |  |  | |  | |
|  | 12 week | 40.92±3.65 | | 37[10] | 37.53±2.64 | 35[18] |  |  |  | |  | |
|  | Score change | 2.96±1.97 | | 0[14] | 2.48±1.72 | 0[13] | 0.055 | 0.859 | 0.111 | | 0.754 | |
| ADHD | Baseline | 39.54±2.73 | | 41[14] | 37.53±2.44 | 43[10] |  |  |  | |  | |
|  | 12 week | 40.30±2.71 | | 42[12] | 42.31±2.03 | 38[13] |  |  |  | |  | |
|  | Score change | 0.77±2.23 | | 0[13] | *4.78±2.16* | *2[11]* | -0.419 | 0.205 | -0.480 | | 0.177 | |
| **Risk avoidance score** | |  | |  |  |  |  |  |  | |  | |
| ASD | Baseline | 41.26±2.88 | | 43[14] | 39.35±3.20 | 43[20] |  |  |  | |  | |
|  | 12 week | 42.20±3.48 | | 42[22] | 41.99±3.19 | 40[22] |  |  |  | |  | |
|  | Score change | 0.93±2.40 | | 0[11] | 2.65±1.85 | 4[8] | -0.175 | 0.574 | -0.093 | | 0.792 | |
| ADHD | Baseline | 39.05±3.09 | | 44[15] | 32.86±3.50 | 39[26] |  |  |  | |  | |
|  | 12 week | 41.49±3.26 | | 40[22] | 37.40±3.29 | 35[21] |  |  |  | |  | |
|  | Score change | 2.44±2.44 | | 0[12] | 4.54±2.83 | 3[18] | -0.181 | 0.582 | 0.186 | | 0.599 | |
| **Achievement score** | |  | |  |  |  |  |  |  | |  | |
| ASD | Baseline | 32.98±2.78 | | 29[19] | 34.14±3.35 | 33[22] |  |  |  | |  | |
|  | 12 week | 33.26±2.29 | | 34[20] | 33.05±3.34 | 34[20] |  |  |  | |  | |
|  | Score change | 0.28±1.73 | | -2[10] | -1.08±2.01 | 0[9] | 0.158 | 0.611 | 0.421 | | 0.239 | |
| ADHD | Baseline | 34.53±2.96 | | 40[19] | 35.79±1.83 | 35[18] |  |  |  | |  | |
|  | 12 week | 39.09±3.42 | | 37[19] | 37.34±2.31 | 35[12] |  |  |  | |  | |
|  | Score change | 4.55±2.26 | | 3[13] | 1.55±1.80 | -1[8] | 0.341 | 0.301 | 0.309 | | 0.383 | |
| **BRUNI test** | |  | | |  | |  |  |  | |  | |
| **Total score** | | | |  |  |  |  |  |  | |  | |
| ASD | Baseline | 14.00±1.62 | | 13[11] | 16.19±2.92 | 18[19] |  |  |  | |  | |
|  | 12 week | 13.24±1.81 | | 13[9] | 16.05±2.84 | 14[16] |  |  |  | |  | |
|  | Score change | -0.76±2.06 | | 0[8] | -0.14±1.69 | 0[9] | -0.072 | 0.817 | -0.150 | | 0.672 | |
| ADHD | Baseline | 15.33±2.70 | | 15[11] | 14.05±1.89 | 15[14] |  |  |  | |  | |
|  | 12 week | 16.28±2.54 | | 9[17] | 15.35±2.24 | 14[15] |  |  |  | |  | |
|  | Score change | -0.94±1.70 | | -2[8] | 1.30±1.75 | 1[9] | -0.297 | 0.266 | -0.303 | | 0.395 | |
| Analyses conducted on an intention-to-treat-basis. Mean±SE. * p<0.01, statistical trend highlighted in italics, intra-group difference by Paired t-test analysis or Wilcoxon signed-ranks test (non-normally distributed variables). Between groups analysis by T-test or Mann Whitney U (non-normally distributed variables). Inter-groups difference analysis by T-test. ANCOVA was used for Inter-group adjusted analysis by sex, age, pharmacological treatment, diagnosis, emotional problems, baseline scores, zBMI, and the quality of the diet. | | | | | | | | | | | | |
